# Accuracy of Colon Capsule Endoscopy for Colorectal Neoplasia Detection in Individuals Referred for a Screening Colonoscopy

**DOI:** 10.1155/2019/5975438

**Published:** 2019-09-03

**Authors:** Michal Voska, Miroslav Zavoral, Tomas Grega, Ondrej Majek, Jan Martinek, Ilja Tacheci, Marek Benes, Gabriela Vojtechova, Pavel Drastich, Jan Bures, Julius Spicak, Barbora Buckova, Ondrej Ngo, Stepan Suchanek

**Affiliations:** ^1^Department of Medicine, First Faculty of Medicine, Charles University, Military University Hospital, Prague, CZ 169 02, Czech Republic; ^2^Institute of Biostatistics and Analyses, Faculty of Medicine, Masaryk University, Brno, CZ 625 00, Czech Republic; ^3^Department of Hepatogastroenterology, Institute for Clinical and Experimental Medicine, Prague, CZ 140 21, Czech Republic; ^4^Charles University in Prague, Institute of Physiology, Czech Republic; ^5^University of Ostrava, Medical Faculty, Czech Republic; ^6^2nd Department of Internal Medicine-Gastroenterology, University Hospital Hradec Kralove, Charles University, Faculty of Medicine in Hradec Kralove, Hradec Kralove, CZ 500 05, Czech Republic; ^7^GEP Clinic, Prague, CZ 130 00, Czech Republic

## Abstract

**Backround:**

Capsule colonoscopy might present an alternative to colonoscopy for colorectal neoplasia screening.

**Aim:**

To assess the accuracy of second-generation capsule colonoscopy (CCE2) for colorectal neoplasia detection compared with conventional colonoscopy (CC).

**Methods:**

From 2011–2015, we performed a multicenter, prospective, cross-over study evaluating the use of CCE2 as a possible colorectal cancer (CRC) screening test based on the assessment of the method's characteristics (accuracy) and safety and patient acceptance of the routine. Enrolled participants fulfilled the CRC screening population criteria if they were asymptomatic, were older than 50, and had no personal or familial history of colorectal neoplasia. The primary outcome was accuracy for the detection of polyps ≥ 6 mm. Secondary outcomes were accuracy for all polyps, polyps ≥ 10 mm, adenomas ≥ 10 mm, and cancers, the quality of bowel cleansing, safety, and CCE2 acceptability by the screening population.

**Results:**

A total of 236 individuals were examined; 11 patients (5%) were excluded. Therefore, 225 subjects (95%) were considered in the intention-to-screen (ITS) group. A total of 201 patients (89%) completed both examinations successfully (per protocol group). In the ITS group, polyps were diagnosed during CC in 114 subjects (51%); polyps ≥ 6 mm, polyps ≥ 10 mm, and adenomas ≥ 10 mm were diagnosed in 34 (15%), 16 (7%), and 11 (5%) patients, respectively. The sensitivity of CCE2 for polyps ≥ 6 mm, polyps ≥ 10 mm, and adenomas ≥ 10 mm was 79% (95% confidence interval (CI): 62–91%), 88% (95% CI: 62–98%), and 100% (95% CI: 72–100%), respectively.

**Conclusion:**

Second-generation capsule colonoscopy is a safe, noninvasive, and sensitive method for colorectal neoplasia detection although CC remains the preferred method for considerable proportion of subjects. CCE2 may therefore be accepted as the primary screening test for colorectal cancer screening.

## 1. Introduction

Colorectal cancer (CRC) is the third most common cancer worldwide: each year, more than 500,000 cancer deaths are associated with this disease [1]. This fact is unfortunate given that CRC is a preventable disease that is curable if diagnosed at early stages [2]. Primary prevention involves modifications to lifestyle and diet. Secondary prevention in asymptomatic individuals (screening) is typically focused on persons aged 50 and older.

The National CRC Screening Program in the Czech Republic was launched in 2000. It is based on a fecal occult blood test (FOBT) offered to asymptomatic individuals aged ≥50 years. In the case of FOBT positivity, a colonoscopy is indicated. The program design was modified at the beginning of 2009 via the introduction of a screening colonoscopy offered to asymptomatic individuals older than 55. However, the participation of the target population in the screening program remains insufficient; in 2011, the participation rate was 25% [3]. According to European recommendations published in 2010, the minimal uptake should be at least 45%, ideally 65% [4]. Therefore, there is a need for other screening modalities with higher acceptance rates. These modalities should adequately detect colorectal neoplasia and still be acceptable by the target population.

Colon capsule endoscopy (capsule colonoscopy; CCE), in which the colorectum is examined via a miniature capsule, is one of the methods that might fulfill the criteria for a reliable and minimally invasive screening method. The primary advantage of CCE is its ability to provide a painless examination with a minimal risk of complications. Colon capsule endoscopy was first used in 2001 (in 2002 in the Czech Republic) to examine the small intestine, and it has become a standard method in this indication [5]. Capsule colonoscopy is based on the same principle but differs in some technical parameters. Second-generation CCE is preferred (CCE2) because it has been equipped with modern technologies.

The goals of this study were as follows: (1) To assess CCE2 accuracy for colorectal neoplasia detection compared with conventional colonoscopy (CC). We investigated the accuracy of CCE2 in terms of detecting polyps ≥ 6 mm (primary outcome), all polyps, polyps ≥ 10 mm, adenomas ≥ 10 mm, and cancers. (2) To assess the safety of CCE2. (3) To assess the acceptance of CCE2 as a primary screening tool among participants.

## 2. Materials and Methods

### 2.1. Design Overview

Individuals referred for a screening colonoscopy were examined prospectively from 2011–2015 at four specialized endoscopy centers (Department of Internal Medicine, 1st Faculty of Medicine Charles University, Military University Hospital, Prague; 2nd Department of Internal Medicine-Gastroenterology, University Hospital in Hradec Kralove; Charles University in Prague, Faculty of Medicine in Hradec Kralove; Department of Hepatogastroenterology, Institute for Clinical and Experimental Medicine, Prague; G.E.P. Clinic, Prague) by both CCE2 and CC.

The enrolled individuals satisfied the following inclusion criteria: they had to be asymptomatic, aged 50–85, free of a family or personal history of colorectal neoplasia, and able to provide informed consent to participate in the study.

We did not include persons with a high risk of CRC (i.e., first-degree family relatives of patients who were diagnosed with CRC at an age younger than 60 years, probands from families with FAP or HNPCC, and individuals with a positive personal history of colorectal neoplasia), individuals with a severe acute ongoing inflammatory bowel disease, persons who had previously undergone surgery for intestinal obstruction, patients with intestinal passage disorder, individuals with severe polymorbidity, and individuals who were anticipated to present noncompliance.

All included participants received oral and written information and provided signed informed consent. The trial was approved by the Ethics Committee of the Military University Hospital Prague (108/8-55/2010).

### 2.2. PillCam COLON 2

Second-generation capsule colonoscopy (PillCam COLON capsule colonoscopy, Given Imaging Ltd., Yoquenam, Israel) is an oval device measuring 31.5 mm × 11.6 mm in size that contains two cameras (one on each side). Each camera can view 172° of the visual field, allowing coverage of nearly 360° of the colon. The capsule is equipped with a battery with a lifetime of approximately 10 hours. To save energy, a special mode called “adaptive frame rate” was developed. The rate of image acquisition varies depending on the movement of the capsule (i.e., 35 images per second in the case of movement and 4 images per second in the case of stagnation). The capsule communicates bidirectionally with electrodes attached to the patient's body at specific locations and a data recorder that hangs around the patient's waist. The data recorder also allows control of the image in real time (real time viewer) and the detection of the small intestine mucosa. After the exam is finished, the data recorder is connected to a workstation and the images are transformed into film sequences that can be viewed using special software.

### 2.3. Capsule Colonoscopy Procedure

All participants underwent bowel preparation based on previously published data [6]. This regimen is similar to that of a colonoscopy based on polyethylene glycol (PEG) solution that is used in the split-dose regimen in combination with low doses of sodium phosphate boosters (Table 1). It enables not only bowel cleansing but also capsule propulsion through the colon.

After capsule ingestion at the endoscopy unit, the position of the capsule was monitored using the data recorder image display. After the capsule passed the duodenum, the participants ingested 30 mL of sodium phosphate solution diluted to 250 mL with water, followed by 1 L of water over the next hour. If the capsule was not excreted within 3 hours of ingesting the first boost, the participants were given a second boost (25 mL sodium phosphate solution diluted in water followed by 0.5 L of water over the next hour). The CCE procedure was considered to be complete when the colon capsule was expelled within 10 hours.

The capsule videos were viewed by investigators who were blinded to the colonoscopy findings. All of the investigators viewing the capsule videos had experience with small-bowel capsules and had received training about viewing colon capsule videos.

Bowel cleansing was evaluated on a 4-point scale for each of the colonic segments (cecum, right colon, transverse colon, left colon, and rectum), and an overall preparation score also was recorded [7]. Polyp size was estimated during capsule video viewing using polyp size estimation software (Given Imaging Ltd.).

### 2.4. Conventional Colonoscopy

**Figure 1:**
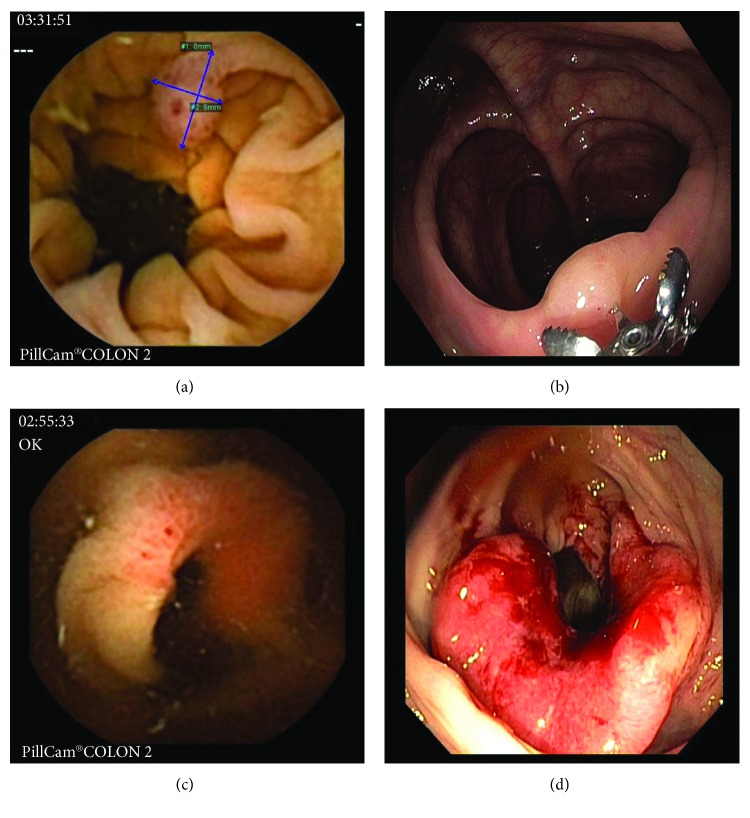
Comparison of the lesions diagnosed with CCE2 and CC ((a) polyp, CCE2; (b) polyp, CC; (c) cancer CCE2; (d) cancer, CC); CCE2: second-generation colon capsule endoscopy; CC: conventional colonoscopy, source: Department of Gastrointestinal Endoscopy, Military University Hospital Prague.

A conventional colonoscopy (Olympus 180 and 190 series colonoscopes) was performed immediately after capsule excretion or, at the latest, 10 hours after capsule ingestion (i.e., the same day as CCE2 in all cases). Colonoscopies were performed by experienced and screening-approved senior endoscopists who were blinded to the results of CCE2. A combination of midazolam (min. 1 mg, max. 5 mg) and fentanyl (min. 50 *μ*g, max. 100 *μ*g) was used together with CO_2_ insufflation in all participants. The bowel preparation quality and number, size, and location of polyps and cancers were recorded. Polyp size was measured by open biopsy forceps during the colonoscopy (Figures 1(a)–1(d)). A second unblinded CC might be performed in cases in which significant CCE2 findings were not detected with CC. Adverse events were assessed as severe (bleeding needing blood transfusion or admission to the hospital, perforation, and capsule retention) and mild or moderate. The acceptability (and preference) of methods was evaluated by a questionnaire that participants completed in the recovery room after both examinations were completed. The most important question the patients answered queried their preferred screening procedure.

### 2.5. Statistical Analysis

In our statistical evaluation of capsule colonoscopy accuracy (with the assumption of CC as the gold standard and the polyps found in the second unblinded CC included), patients with polyps ≥ 6 mm in the CC were considered to be positive. If polyps were found in both CC and CCE2, the size-matching finding with CC was considered in the per-patient analysis if the CCE2 maximum size was within a 50% margin of error around the CC maximum size. If no polyp was found with CC (including a possible second unblinded CC) and polyps ≥ 6 mm were found at CCE2, CCE2 was considered to be a false positive. The statistical analysis was performed using the program Stata/IC 13.1 (StataCorp, College Station, TX, USA).

Moreover, in a secondary effectiveness analysis, we estimated “practical accuracy,” where we assumed that CCE2 was performed as a prescreening test before a definitive CC examination. Second-generation capsule colonoscopy was considered to be positive (i.e., indicating the need for a follow-up colonoscopy referral) if either an incomplete or a polyp larger than the specific cut-off was detected. As opposed to the primary analysis, we applied no margin-of-error correction. As a cut-off, values between 1 and 6 mm were considered. We next constructed a receiver operating characteristic (ROC) curve to visually identify promising cut-off points for CC referral. The sensitivity and specificity were estimated considering adenomas ≥ 6 mm at CC as the endpoint.

## 3. Results

**Figure 2:**
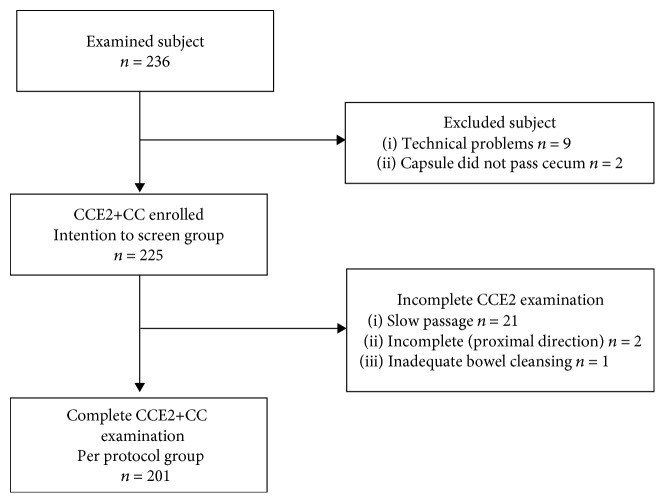
STARD flowchart (STAndards for the Reporting of Diagnostic accuracy studies) of the patients' enrollment in the study. CCE2: second-generation colon capsule endoscopy; CC: conventional colonoscopy.

In total, 236 individuals were examined by both methods (CCE2 and CC). All of the colonoscopies were complete. Nine individuals were excluded from the evaluation because of technical problems (signal interference); the capsule did not pass the cecum within 12 hours in 2 individuals. Therefore, 225 subjects (95%) were considered to fall within the intention-to-screen group (120 men, 53%; 105 women, 47%; mean age: 59 years; range: 50–81 years; Table 2). Slow passage (more than 10 hours) was reported in 21 individuals. A total of 201 patients (89%) completed both examinations successfully with the capsule egested within 10 hours (per protocol group; see the STARD (STAndards for the Reporting of Diagnostic accuracy studies) flowchart in Figure 2). The average transit time of the capsule through the digestive tract was 3 hours and 48 minutes. The capsule colon transit time was reported to be fewer than 45 minutes in 34 participants (15%). Nevertheless, we included these patients in the analysis. Capsule retention (≥14 days) was not reported. The results were evaluated for the intention-to-screen group.

In the intention-to-screen analysis (*N* = 225 patients), polyps were diagnosed during CC in 114 persons (51%), and polyps ≥ 6 mm, polyps ≥ 10 mm, all adenomas, and adenomas ≥ 10 mm were diagnosed in 34 (15%), 16 (7%), 59 (26%), and 11 (5%) patients, respectively. The sensitivity of CCE2 to all polyps, polyps ≥ 6 mm, polyps ≥ 10 mm, all adenomas, and adenomas ≥ 10 mm was 82%, 79%, 88%, 81%, and 100%, respectively. The specificity for all polyps, polyps ≥ 6 mm, and polyps ≥ 10 mm reached was 86%, 97%, and 99%, respectively. The specificity of adenomas was not evaluated because CCE2 does not allow polyp retrieval and histopathology assessment. Two cancers found in the study were diagnosed using both methods. Detailed results of the accuracy with confidence intervals are summarized in Table 3.

Adequate colon cleansing (A, B) was achieved in 90% (CCE2) and 94% (CC) of individuals (Table 4). The difference between colon cleansing with CCE2 or CC was not statistically significant (McNemar test *p* = 0.08).

No serious adverse events were registered; mild adverse events in 6 subjects (3%) were associated with bowel preparation (*N* = 2; emesis, vertigo) and the colonoscopy (*N* = 4; abdominal pain).

According to patient satisfaction surveys, 105 individuals (47%) preferred CCE2 as the primary screening test; 120 individuals (53%) preferred CC as the only screening modality.

**Figure 3:**
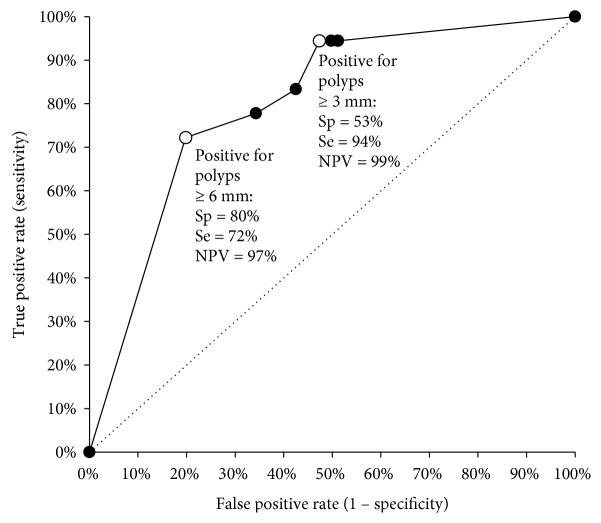
ROC curve comparing false-positive and true-positive rates (considering confirmed adenomas larger than 6 mm as the endpoint) at different levels of CCE2 polyp size cut-offs for referral to OC. Sp: specificity, Se: sensitivity, NPV: negative predictive value.

Figure 3 shows the ROC curve for our secondary analysis of “practical accuracy” that aimed at detecting adenomas ≥ 6 mm. With a high-sensitivity cut-off point (follow-up CC referral for patients with polyps ≥ 3 mm at CCE2), the estimated sensitivity was 94% and the negative predictive value was 99%. As a result, there was a very low risk of significant neoplasia despite a negative result (i.e., a high negative predictive value). With a high-specificity cut-off point (follow-up CC referral for polyps ≥ 6 mm at CCE2), the estimated sensitivity was 72% and the negative predictive value was 97%. The data in Table 5 reveal that offering the choice of screening test (CC or CCE2 with the high-specificity cut-off) to a cohort of 10,000 individuals willing to undergo a screening examination leads to a decrease in the number of CCs by 2,303 (out of 10,000); 16 patients with polyps ≥ 6 mm will be missed (out of 800).

## 4. Discussion

Colorectal cancer is not only a serious health condition but also a socioeconomic problem. Previously published work confirmed the effectiveness of established screening methods, such as fecal occult blood tests, guaiac-based [8] or immunochemical (FIT) [9], flexible sigmoidoscopy [10], and colonoscopy [11], which is still regarded as the gold-standard method.

Capsule colonoscopy is a relatively new tool; the first comprehensive data related to this technique were published in 2009 [12]. The overall sensitivity of the first-generation capsule was not good enough for implementing this method as a primary screening method.

Three principal studies have assessed the effectiveness of the second generation of capsule colonoscopy (CCE2) in a screening population [13–15]. Compared with CCE1 results, all three studies demonstrated a higher sensitivity (89%, 87%, and 85%) and specificity (96%, 92%, and 97%) for CCE2 for clinically relevant neoplasia (i.e., polyps larger than 10 mm). The Czech study confirmed the higher efficiency of CCE2 compared with CCE1 at detecting colorectal neoplasia. The sensitivity and specificity for polyps and adenomas of all sizes were higher than that of CCE1 [12]. Compared with the CCE2 studies noted above, the sensitivity and specificity of this technique in our study to polyps ≥ 10 mm were similar (88% and 99%, respectively).

The higher accuracy of CCE2 compared with CCE1 may be explained by the technological developments associated with CCE2. The second-generation technology has a larger field of vision; both cameras together produce a nearly 360° field of view. Another advantage of CCE2 is the variable number of recorded pictures (i.e., the adaptive frame rate), which saves battery life and prolongs the time of the procedure. Therefore, it increases the probability that the capsule will examine the entire colon and rectum. Next, positive technological changes have resulted in the emergence of a new type of data recorder that communicates bidirectionally with the capsule and enables real-time observations. The capsule delay can be monitored and managed using prokinetic agents. The data recorder recognizes the first small intestine mucosa images and alerts the patient with vibrations so that the first booster can be administered.

Concerning the technical aspects of the procedure, the only issue that needed to be overcome in our study was the dearth of pictures, which occurred during the first examinations (i.e., gaps). This problem was caused by interference with the telemetric monitoring of patients with cardiac disease observed in the same building. As a result, 9 patients with incomplete records had to be excluded from the study evaluation. This problem was solved by new CCE2 software that was regularly updated.

The prevalence of adenomatous polyps (26%) in our study was nearly the same as that in the Czech National CRC Screening Program, which reported a 27% adenoma detection rate (2013 Screening Colonoscopies Database) [16].

Any primary screening tool needs to be accepted by the screening population. Therefore, all subjects in our study answered the question about their test preference (CCE2 versus CC) if only one screening procedure was to be repeated. Participants were informed that CCE2 was the first method in the two-step program with a lower accuracy (known from previously published data) than CC. Furthermore, they were informed about the need for CC in the case of finding a polyp. One hundred and five subjects (47%) preferred the colon capsule as the primary screening test, and 120 subjects (53%) preferred optical colonoscopy as the only screening modality.

Our study reported a lower acceptance rate of CCE2 compared with previously published studies [17, 18]. Many participants who refuse CC do so to avoid the preparation more than the procedure, which is usually performed under sedation. The necessity of a second bowel cleansing and CC in the case of a positive finding on CCE2 is likely the reason why more than a half of participants prefer CC to CCE2, even if it only applies to only 20–30% of screened patients.

We are aware of a bias, because the questionnaire was completed in the recovery room two hours after conscious sedation. This should be ideally the day after. In addition, the study design does not reflect a real-world scenario, where CCE would be a procedure performed at home.

In our secondary analysis, we visualized the relationship between the diagnostic threshold for CC referral and CCE2 accuracy characteristics. We identified two (high-sensitivity and high-specificity) thresholds for possible utilization of CCE2 as a CRC screening filter test. Although we acknowledge the rather crude manner of this exercise, it demonstrates that future fine-tuning of CCE2 use in clinical practice—concerning its accuracy, effectiveness, and cost-effectiveness—is warranted. Our model analysis employing high-specificity settings suggests that the ability to choose CCE2 rather than CC may lead to a significant decrease in the number of CCs needed at the cost of a few more patients with missed adenomas. However, there is currently a concern regarding colonoscopy service capacity and cost. As a result, the potential value of including CCE2 in the screening algorithm in different settings should be ascertained.

Second-generation capsule colonoscopy can also be used for colonoscopy selection in individuals with positive FIT. Such an approach was adopted by an Irish group [14]. Quantitative FIT with a 100 ng Hb/mL cut-off level was used; in the case of test positivity, CCE2 examination followed. Sixty-two individuals were included, and a 71% reduction in colonoscopies was achieved. However, these results are not transferrable to the Czech population. Another type of FIT is being used in the Czech Republic, where the prevalence of colorectal neoplasia differs from that in Ireland. An ongoing Czech multicenter study is examining individuals with positive semiquantitative FIT [19]. Its objective is to show that the negative predictive value of CCE2 applied in patients with positive FIT test results is sufficient to safely spare patients from an optical colonoscopy examination.

Second-generation capsule colonoscopy has its limitations. The accuracy of the method largely depends on bowel cleanliness. Split regimens based on polyethylene glycol with additional booster preparations are necessary to obtain adequate bowel cleanliness. Second, excreting the capsule within its battery life is an important factor for effective CCE2-based examination. The reported 90% excretion rate was lower than the recommended 95% cecal intubation rate in screening colonoscopies. Third, patients with positive findings have to undergo another bowel preparation. The ability to perform a colonoscopy immediately after CCE2 may cement the advantage of using the same preparation regimen.

## 5. Conclusion

Colonoscopy remains the gold-standard procedure for CRC screening programs. Second-generation capsule colonoscopy represents an alternative method with sufficient sensitivity for colorectal neoplasia detection. Future research efforts should focus on improved bowel preparation as well as on the cost effectiveness of incorporating CCE2 into standard CRC screening practices.

## Figures and Tables

**Table 1:** Bowel preparation regimen.

	Schedule	Preparation
Day -2	All day	Low-residue diet, abundant liquids
Day -1	All day	Clear liquids
	7 pm–9 pm	3 litres of PEG
Examination day	7 am–8:30 am	1 litres of PEG
	1 hour after PEG	Swallow capsule^∗^
	1st booster (detection of the small intestine)	30 mL of NAP + 1.0 litres water
	2nd booster (3 hours after the first booster)	25 mL of NAP + 0.5 litres water
	Suppository (2 hours after the second booster)	Glycerin suppository 2 g

^∗^Administered prokinetic agents (metoclopramide, 10 mg), if capsule in the stomach > 1 h; PEG: polyethylene glycol; NAP: natrium phosphate.

**Table 2:** Clinical characteristics of analysed individuals.

Age	Men	Women	Total
*N*	120 (53%)	105 (47%)	225
Mean	59	59	59
Minimum	50	50	50
Maximum	81	77	81

**Table 3:** Accuracy of CCE2 in colorectal neoplasia detection (per-patient analysis, intention-to-screen group, *N* = 225).

	Colonoscopy		Colonic capsule					
	Prevalence (number; proportion)		Sensitivity (number of matching outcomes; proportion among CC+; CI)			Specificity (number of matching outcomes; proportion among CC-; CI)		
Polyp	114	51%	94	82%	CI: 74-89%	96	86%	CI: 79-92%
≥6 mm	34	15%	27	79%	CI: 62-91%	186	97%	CI: 94-99%
≥10 mm	16	7%	14	88%	CI: 62-98%	207	99%	CI: 97-100%
Adenoma	59	26%	48	81%	CI: 69-90%	—	—	—
≥10 mm	11	5%	11	100%	CI: 72-100%	210	98%	CI: 95-99%
Carcinoma	2	1%	2	100%	—	223	100%	—

CCE2: second generation of colon capsule endoscopy; CI: 95% confidence interval.

**Table 4:** Level of bowel preparation (CCE2 and CC, *N* = 225).

		Number (% among reported) by segment					
Method	Cleansing	Cecum	Ascending	Transverse	Descending	Rectum	Total
CCE2	A	90 (42%)	91 (48%)	94 (50%)	96 (51%)	90 (49%)	98 (44%)
	B	96 (45%)	78 (41%)	77 (41%)	69 (37%)	68 (37%)	104 (46%)
	C	23 (11%)	15 (8%)	11 (6%)	18 (10%)	22 (12%)	16 (7%)
	D	5 (2%)	4 (2%)	5 (3%)	4 (2%)	4 (2%)	7 (3%)
	Not reported	11	37	38	38	41	
CC	A	106 (49%)	91 (50%)	101 (55%)	100 (54%)	102 (56%)	111 (49%)
	B	93 (43%)	79 (43%)	72 (39%)	74 (40%)	72 (39%)	100 (44%)
	C	13 (6%)	10 (5%)	7 (4%)	7 (4%)	6 (3%)	10 (4%)
	D	4 (2%)	3 (2%)	3 (2%)	3 (2%)	3 (2%)	4 (2%)
	Not reported	9	42	42	41	42	

**Table 5:** Simulation model comparing the outcomes for a cohort of 10,000 individuals undergoing either screening colonoscopy (CC) only or the choice of the screening test of CC or CCE2 (high-specificity cut-off). Model parameters (patient preference, CCE2 positivity, and pre- and post-CCE2 adenoma prevalence) were taken from this study.

CC only				CC screening			
				No. of patients	Adenoma ≥ 6 mm		
Screening CC				10,000	800 (8%)		

Patient choice		CCE2 prescreening		CC screening			
	Preference	No. of patients	Positive	No. of patients	Adenoma ≥ 6 mm	Decrease in CCs	Missed adenomas
Screening CC	53%			5300	424 (8%)	2303	16
Prescreening CCE2	47%	4700	2397 (51%)	2397	360 (15%)		

## Data Availability

The data used to support the findings of this study are included within the article.
